# Star-Shaped Polydimethylsiloxanes with Organocyclotetrasilsesquioxane Branching-Out Centers: Synthesis and Properties

**DOI:** 10.3390/polym14020285

**Published:** 2022-01-12

**Authors:** Yulia S. Dyuzhikova, Anton A. Anisimov, Alexander S. Peregudov, Mikhail I. Buzin, Galina G. Nikiforova, Viktor G. Vasil’ev, Sergey A. Kostrov, Alexander I. Buzin, Alexei A. Stupnikov, Yulia N. Malakhova, Olga I. Shchegolikhina, Aziz M. Muzafarov

**Affiliations:** 1 Institute of Organoelement Compounds of the Russian, Academy of Sciences, Vavilov St. 28, 119991 Moscow, Russia; vysochinskaja@yandex.ru (Y.S.D.); asp@ineos.ac.ru (A.S.P.); buzin@ineos.ac.ru (M.I.B.); ggn@ineos.ac.ru (G.G.N.); viktor@ineos.ac.ru (V.G.V.); sergeykostrov1996@gmail.com (S.A.K.); olga@ineos.ac.ru (O.I.S.); aziz@ispm.ru (A.M.M.); 2 Department of Physics, Moscow State University, Leninskie Gory 1-2, 119991 Moscow, Russia; 3 Institute of Synthetic Polymeric Materials of Russian, Academy of Sciences, Profsoyuznaya St. 70, 117393 Moscow, Russia; al37919@gmail.com; 4 National Research Center “Kurchatov Institute”, Akademika Kurchatova Square. 1, 123182 Moscow, Russia; alexei.stupnikov@mail.ru (A.A.S.); j.malakhova@mail.ru (Y.N.M.); 5 M. V. Lomonosov Institute of Fine Chemical Technologies, MIREA–Russian Technological University, Prospekt Vernadskogo 86, 119571 Moscow, Russia

**Keywords:** star-shaped polymer, polydimethylsiloxane, organocyclotetrasilsesquioxane

## Abstract

New non-crystallizable low-dispersity star-shaped polydimethylsiloxanes (PDMS) containing stereoregular *cis*-tetra(organo)(dimethylsiloxy)cyclotetrasiloxanes containing methyl-, tolyl- and phenyl-substituents at silicon atoms and the mixture of four stereoisomers of tetra[phenyl(dimethylsiloxy)]cyclotetrasiloxane as the cores were synthesized. Their thermal and viscous properties were studied. All synthesized compounds were characterized by a complex of physicochemical analysis methods: nuclear magnetic resonance (NMR), FT-IR spectroscopy, gel permeation chromatography (GPC), thermogravimetric analysis (TGA), differential scanning calorimetry (DSC), viscometry in solution, rheometry, and Langmuir trough study.

## 1. Introduction

Creation of polymers with new macromolecular architecture is one of the principal driving forces in the development of polymer science. The structure–properties relationship, which is the cornerstone of polymeric chemistry guarantees that polymer of unusual architecture would possess an unusual complex of properties [1,2]. Among a vast variety of macromolecular structures, a big class of branched high-molecular compounds is distinguished. Bright representatives of this class are the star-shaped polymers **(SSP)**. These are branched macromolecules in which the arms (linear polymers) ‘grow’ from one branching center (core). So, an atom, a molecule or a macromolecule can act as the branching center. At the same time, it is supposed that the length of arms is identical [3]. An important parameter for such polymers is the number of arms, their functionality and molecular weight. The main feature of **SSPs** distinguishing them from linear analogs of identical molecular masses is their compact structure (smaller hydrodynamic volume, and, therefore, less viscosity) and possible wider functionality [4,5,6].

At present, there are various approaches to **SSP** synthesis. They can be divided into three main types: “core-first”, “arm-first”, and “grafting-onto” [6]. Each of these approaches has a certain number of advantages and disadvantages.

Our interest in synthesis and research of siloxane **SSP** properties is determined by their valuable properties, such as stability at high and low temperatures, water repellency, biocompatibility, high gas permeability, UF radio-resistance, and a number of other unique characteristics [7,8,9,10]. So far, the majority of the available publications on **SSP** synthesis describe polymers where only one component has siloxane nature—it is either arms [11,12,13,14,15,16,17,18,19,20], or a core [21,22,23,24,25,26,27,28,29,30,31,32,33,34,35,36,37]. The synthesis and study of properties of totally siloxane star-shaped systems [38,39,40,41,42] are described in only some of publications.

We chose the grafting-onto method for the formation of star-shaped siloxane polymers. This approach allows high-molecular compounds with the highest level of structural control to be obtained, since **SSP** core and arms can be synthesized and characterized separately, even before obtaining a target product. By using this method, we synthesized a series of low dispersity polydimethylsiloxane **SSPs**. Their molecules contain flexible stereoregular phenylcyclosilsesquioxanes [PhSi(O)OSiMe_2_H]_n_ of various sizes, structures, and functionality (*n* = 4, 5, 6, 8, 12), such as the branching center (core), as well as the cage octahedral silsesquioxane playing the role of rigid point center [43,44]. It should be noted that only two of six obtained polymers had three-dimensional spatial structures, traditional for **SSP**, in which the arms spread in all directions from the branching center. These polymers contain tris-*cis*-tris-*trans*-dodeca[phenyl(dimethylsiloxy)]cyclododecasiloxane and octakis[(dimethylsiloxy)]octasilsesquioxane as the branching center. Other four polymers with the molecules containing *cis*-configuration cycles as the core had absolutely different spatial geometry, where all polydimethylsiloxane arms were arranged on one side of the branching center plane. Similar star-shaped structures were not known before. In our previous publication, we assessed the influence of size, branching cyclosiloxane center and arms number on the properties of **SSPs** on their basis. In this paper, we were interested in the influence of the frame in cyclosiloxane core, and also the stereoregularity of arms arrangement relative to the cycle plane. For objective comparison, we obtained the mixture of four stereoisomers of tetra[phenyl(dimethylsiloxy)]cyclotetrasiloxane, and stereoregular cyclotetrasilsesquioxanes, containing methyl-, tolyl-, and phenyl-substituents at silicon atom. The synthesized cyclotetrasilsesquioxanes were used as cores for **SSP** synthesis.

## 2. Experimental Part

### 2.1. Materials

Solvents were prepared according to earlier described technique [45].

*n*-BuLi (1.6 M solution in hexane), vinyldimethylchlorosilane—commercial products (Acros). Hexamethylcyclotrisiloxane (D_3_); Karsted’s catalyst (solution of platinum complex (0) with 1,3-divinyl-1,1,3,3-tetramethyldisiloxane in a xylene, Pt~2%mass.)—commercial products (Sigma-Aldrich, St. Louis, MI, USA); sulfocationite (Amberlyst 15)—a commercial product (abcr, Karlsruhe, Germany).

Functional stereoregular organocyclosilsesquioxanes were synthesized according to earlier described technique [46,47,48,49,50].

### 2.2. Methods

NMR spectra were registered on Bruker Avance™ 600 spectrometer (Bruker, Berlin, Germany) operating at 600.22, 150.93 and 119.26 MHz for ^1^H, ^13^C, and ^29^Si cores, respectively, and Bruker Avance II 300 (Bruker). ^1^H and ^13^C chemical shifts were measured relative to residual signals of corresponding solvents and calculated to tetramethylsilane. ^29^Si chemical shifts were measured relative to external standard - tetramethylsilane.

IR-spectra were registered with the use of FT-IR spectrometer Bruker Tensor 37 (Bruker, Berlin, Germany). Samples were prepared by pressing KBr pellets.

High-resolution mass spectra (HRMS) were measured using Bruker micrOTOF II instrument with electrospray ionization (ESI) (Bruker, Berlin, Germany).

The analysis by gel permeation chromatography (GPC) method was carried out on chromatographs “Shimadzu” (Kyoto, Japan), detector—RID refractometer-20 A, column—PSS SDV analytical 100 000 A (size (300 × 8 mm)); eluent—toluene; “Shimadzu” (Kyoto, Japan), detectors—RID refractometer-20 A and photodiode detector SPD-M20A, column—Phenogel 500 A (the size (300 × 7,8 mm)); eluent—tetrahydrofuran.

The study by DSC method was conducted on DSC-822e device (Mettler-Toledo, Greifensee, Switzerland) at 10 °C/min heating and cooling rates.

The study by TGA method was conducted on Derivatograph-C device, (MOM, Mateszalka, Hungary) in air and in argon at 10 °C/min heating rate.

Rheological studies were conducted on Anton Paar MCR 302 rheometer (Graz, Austria), in the mode of constant shear rate, plane–plane measuring mode, plane diameter 25 mm.

The reduced viscosity of diluted solutions of obtained polymers was measured with an Ubbelohde suspended level capillary viscometer in the concentrations range of 0.25–1 dl/g at 25 ± 0.05 °C.

Formation and study of Langmuir layer properties was carried out on Minitrough Extended (KSV, Espoo, Finland) with maximum area of the interphase surface equal to 558 cm^2^. Compression and expansion speed was 15 cm^2^ min^−1^. As a subphase, purified and demineralized water with a specific resistance of 18.2 MOhm cm (at 25 °C) thermostatically maintained at 20 °C with the use of Milli-Q (Millipore, Burlington, MA, USA) integrated water purification system was utilized. The studied star-shaped copolymers were dissolved in chloroform. Surface pressure was measured by Wilhelmy’s method with the use of a rough platinum plate with 0.1 mN m^−1^ accuracy. Surface potential was measured by method of vibrating electrode (KSV, Espoo, Finland) with 1 mV accuracy. The Langmuir layers morphology directly on water surface was visualized by Brewster angle microscope BAM-300 (KSV, Espoo, Finland). The images obtained, corresponding to 200 × 200 μm^2^ interface surface, were geometrically corrected taking into account Brewster angle of water (53.1°). Confidence intervals for the values obtained from surface pressure and surface potential isotherms are 0.1 mN m^−1^ for surface pressure, 30 Å^2^ for the area per a molecule, 0.3 Å^2^ for the area per unit of a dimethylsiloxane and 5 mV for surface potential.

#### 2.2.1. Reaction of Cis-Tetra[phenyl(dimethylsiloxy)cyclotetrasiloxane Isomerization

Briefly, 0.3 g (0.4 mmol) of *cis*-tetra[phenyl(dimethylsiloxy)]cyclotetrasiloxane and 0.015 g (5 masses. %) of sulfocationic resin were loaded into a two-neck flask. The reaction in the ultrasonic bath continued for 4 h at 70 °C. To remove the sulfocationite, the reaction mass was dissolved in hexane and filtered through the paper filter. The yield: 0.27 g (90%) after solvent removing

**^1^H NMR** (600 MHz, CDCl_3_, ppm): δ 7.87–7.19 (m, Ph), 4.98–4.61 (m, SiH), 0.37–0.02 (m, Si(CH_3_)_2_), 7.21–7.23.

**^13^C NMR** (150 MHz, CDCl_3_, ppm): δ 0.15, 0.23, 0.35, 0.44, 0.54, 0.63, 127.56, 127.60, 127.63, 127.67, 127.70, 127.74, 130.0, 130.05, 130.10, 130.15, 130.22, 130.26, 132.47, 132.51, 132.68, 132.71, 132.90, 132.98, 134.0, 134.06, 134.09, 134.15, 134.18, 134.22.

**^29^Si NMR** (119 MHz, CDCl_3_, ppm): δ-78.46,-78.35,-78.33,-3.91,-3.83,-3.79,-3.75,-3.68,-3.66.

**IR** (ν/cm^−1^): 3095, 3073, 3053, 3016, 3007, 2961, 2902, 2134, 1430, 1253, 1135, 1066, 900, 836, 771, 697, 594, 485.

**Mass spectrometry** (ESI) of m/z is calculated for: C_32_H_52_NO_8_Si_8_, [(M + NH_4_)^+^]: 802.18, found 802.1842.

**Elemental analysis** found, %: C, 49.18; H, 6.15; Si, 28.35. Calculated for C_32_H_48_O_8_Si_8_, %: C, 48.94; H, 6.16; Si, 28.61.

#### 2.2.2. Synthesis of PDMS-15

Briefly, 132 mL of hexane, 45.06 g (202.5 mmol) of D _3_ and 18.1 mL of *n*-BuLi (28.9 mmol, 1.6 M solution in hexane) were loaded into a one-neck flask supplied with a magnetic stirrer. In 12 h, 75 mL of tetramethylene oxide (THF) was mixed into the system. In 6 h after adding THF, 7 g (57.9 mmol) of vinyldimethylchlorosilane was added dropwise. The reaction mass was filtered off from LiCl through the paper filter and solvents were removed to constant weight. The yield: 42.85 g (95%) of a viscous transparent liquid.

**^1^H NMR** (600 MHz, CDCl_3_, ppm): 6.25–5.75 (m, SiVin); 1.41–1.32 (m, SiBu); 0.96–0.92 (m, SiBu); 0.62–0.57 (m, SiBu); 0.21–0.10 (m, Si (CH_3_)_2_).

**IR** (ν/cm^−1^): 3052, 2963, 2905, 2875, 2860, 2799, 1944, 1596, 1445, 1410, 1260, 1191, 1092, 1022, 958, 862, 798, 702, 687, 669, 517.

**GPC**: M_n_ = 2.4 kDa, PDI = 1.13.

#### 2.2.3. Synthesis of Star-Shaped Siloxane Polymers

##### General Synthesis Technique

Toluene, organocyclotetrasilsesquioxane, **PDMS-15** and Karsted’s catalyst were loaded into a one-neck flask supplied with a magnetic stirrer. Stirring continued for 2 days. Then toluene solution was filtered through silica gel to remove Pt and concentrated. All polymers were purified by method of preparative chromatography.

#### 2.2.4. Synthesis of Me_4_-15 Star-Shaped Polymer

Toluene (22 mL), *cis*-tetra[methyl(dimethylsiloxy)]cyclotetrasiloxane (0.2 g, 0.38 mmol), **PDMS-15** (2 g, 1.6 mmol) and Karsted’s catalyst (4 µL). The yield: 1.99 g (91%) of a viscous transparent liquid.

**^1^H NMR** (600 MHz, CDCl_3_, ppm): δ 1.36–1.31 (m, SiBu); 0.92–0.89 (m, SiBu); 0.57–0.54 (m, SiBu); 0.49–0.44 (m, SiCH_2_ CH_2_); 0.16–0.06 (m, Si(CH_3_)_2_, SiCH_3_).

**IR** (ν/cm^−1^): 2963, 2911, 2801, 2054, 1946, 1732, 1602, 1447, 1408, 1261, 1090, 1028, 799, 695.

**GPC**: M_n_ = 6.6 kDa, PDI = 1.08.

#### 2.2.5. Synthesis of Ph_4_-15 Star-Shaped Polymer

Toluene (23 mL), *cis*-tetra[phenyl(dimethylsiloxy)]cyclotetrasiloxane (0.3 g, 0.38 mmol), **PDMS-15** (2 g, 1.6 mmol) and Karsted’s catalyst (4 µL). The yield: 2.11 g (96%) of a viscous transparent liquid.

**^1^H NMR** (600 MHz, CDCl_3_, ppm): δ 7.30–7.24 (m, SiPh); 7.09–7.06 (m, SiPh); 1.37–1.30 (m, SiBu); 0.92–0.89 (m, SiBu); 0.57–0.54 (m, SiBu); 0.50–0.39 (m, SiCH_2_CH_2_); 0.21–0.02 (m, Si(CH_3)2_).

**IR** (ν/cm^−1^): 2962, 2910, 1409, 1260, 1091, 1027, 801, 740, 697.

**GPC**: M_n_ = 6.7 kDa, PDI = 1.14.

#### 2.2.6. Synthesis of Ph^r^_4_-15 Star-Shaped Polymer

Toluene (23 mL), mixture of tetra[phenyl(dimethylsiloxy)]cyclotetrasiloxane (0.32 g, 0.41 mmol) isomers, **PDMS-15** (2 g, 1.6 mmol) and Karsted’s catalyst (4 µL). The yield: 2 g (91%) of a viscous transparent liquid.

**^1^H NMR** (600 MHz, CDCl_3_, ppm): δ 7.77–7.04 (m, SiPh); 1.34–1.24 (m, SiBu); 0.88–0.85 (m, SiBu); 0.54–0.50 (m, SiBu); 0.42–0.25 (m, SiCH_2_CH_2_); 0.21–0.21 (m, Si(CH_3_)_2_).

**IR** (ν/cm^−1^): 3062, 2962, 2910, 2800, 1409, 1260, 1088, 1027, 800, 739, 697.

**GPC**: M_n_ = 6 kDa, PDI = 1.11.

#### 2.2.7. Synthesis of Tol_4_-15 Star-Shaped Polymer

Toluene (42 mL), *cis*-tetra[tolyl(dimethylsiloxy)]cyclotetrasiloxane (0.58 g, 0.7 mmol), **PDMS-15** (3.84 g, 3.1 mmol) and Karsted’s catalyst (6 mcl). The: 3.95 g (98%) of a viscous transparent liquid.

**^1^H NMR** (600 MHz, CDCl_3_, ppm): δ 7.21–7.18 (m, SiTol); 6.69–6.89 (m, SiTol); 6.69–6.89 (m, SiTol); 1.36–1.30 (m, SiBu); 0.91–0.89 (m, SiBu); 0.57–0.54 (m, SiBu); 0.47–0.37 (m, SiCH_2_CH_2_); 0.19–0.02 (m, Si(CH_3_)_2_).

**IR** (ν/cm^−1^): 2962, 2912, 2800, 1408, 1260, 1091, 1027, 800, 690.

**GPC:** M_n =_ 7.9 kDa_,_ PDI = 1.13.

## 3. Results and their Discussion

### 3.1. Cores Synthesis

**Scheme 1:**
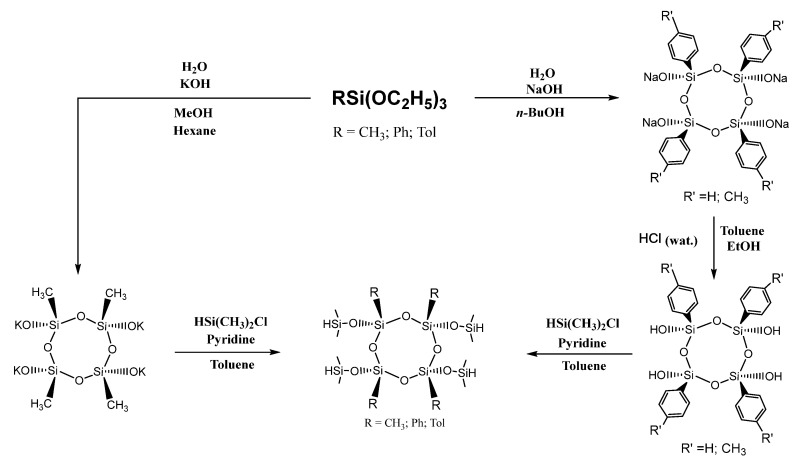
General scheme of *cis*-tetraorganocyclotetrasilsesquioxanes synthesis.

Synthesis of *cis*-tetraorganocyclotetrasilsesquioxanes containing methyl-, tolyl-, and phenyl- groups was carried out according to Scheme 1.

The respective organotrialkoxysilane was treated by equimolar quantity of sodium hydroxide or potassium hydroxide in presence of equimolar amount of water. *n*-Butanol was used as solvent in case of tolyl- and phenyl- substituent; in case of methyl- substituent, methanol and hexane mixture with 1/7 ratio was used. For further use of these cycles as **SSP** cores, they were treated by dimethylchlorosilane according to Scheme 1.

**Scheme 2:**
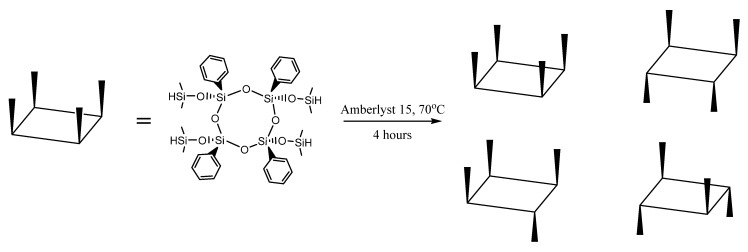
Scheme of *cis*-tetra[phenyl(dimethylsiloxy)]cyclotetrasiloxane isomerization.

The isomerization of *cis*-tetra[phenyl(dimethylsiloxy)]cyclotetrasiloxane was carried out in mass in presence of sulfocationite (Amberlyst 15) at 70 °C for 4 h (Scheme 2). As a result, the mixture of all four isomers in equal quantities with 90% yield was formed.

**Figure 1:**
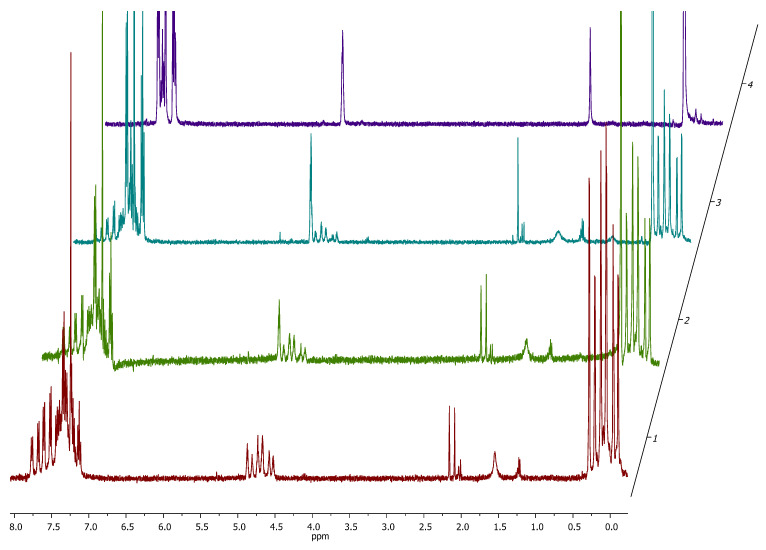
^1^H NMR-spectra of initial compound (violet), after 1 h from the beginning of reaction (turquoise), after 2 h (green) and after 4 h (burgundy).

The kinetics of isomerization process was monitored by ^1^H NMR method (Figure 1).

Reaction mass samples were taken after 1, 2, and 4 h from the beginning of reaction.

According to the ^1^H NMR spectroscopy data of initial compound, *cis*-tetra[phenyl (dimethylsiloxy)]cyclotetrasiloxane (a violet curve), in the field of 4.9 ppm we observe a signal that corresponds to SiH-group. As isomerization goes on, the emergence of new signals in the field of 4.5–5 ppm (turquoise and green curves), which correspond to SiH isomers groups is observed. The appearance of signals of equal intensity for all SiHgroups in the region of 4.5–5 ppm (burgundy curve) shows the moment when the reaction ends. The isomerization reaction is completely over (stopped) 4 h after the start.

**Figure 2:**
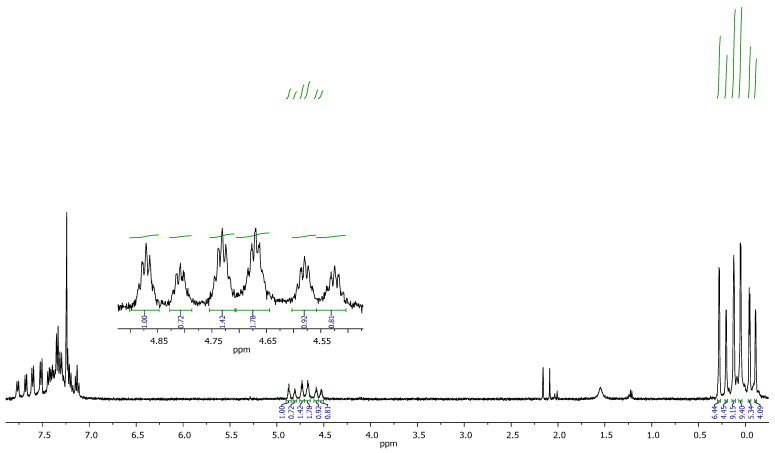
^1^H NMR-spectrum of isomers mixture.

Composition of mixture obtained and structure of isomers were identified by ^1^H NMR-spectroscopy (Figure 2).

**Figure 3:**
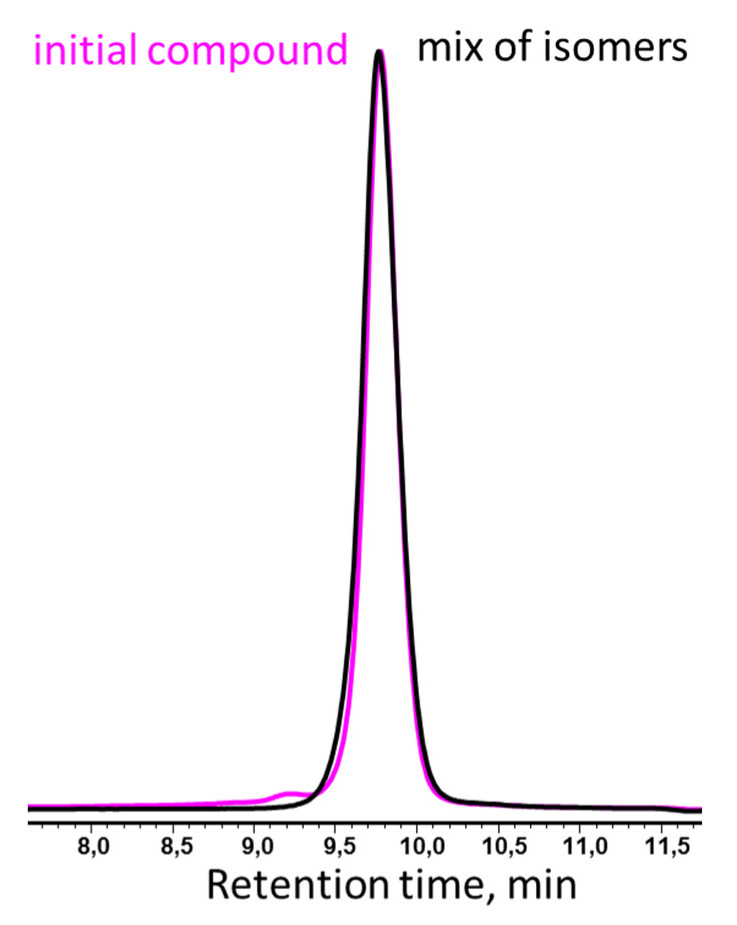
GPC curves of the initial *cis*-tetra[phenyl(dimethylsiloxi)]cyclotetrasiloxane and its mix of isomers.

According to GPC data, the hydrodynamic radius of isomers in the mixture and of initial *cis*-tetra[phenyl(dimethylsiloxy)]cyclotetrasiloxane coincide (Figure 3).

### 3.2. Synthesis of Arm

**Scheme 3:**
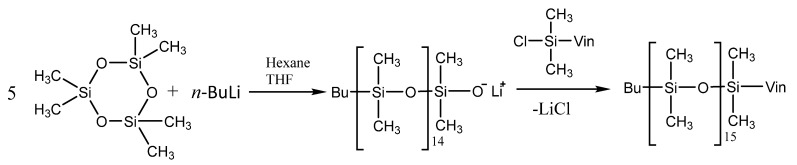
Scheme of PDMS-arm synthesis.

The monofunctional PDMS-arm with *n* = 15 polymerization degree was synthesized by method of living anionic polymerization of hexamethylcyclotrisiloxane in presence of *n*-BuLi, with subsequent blocking by vinyldimethylchlorosilane (Scheme 3).

### 3.3. Assembly of Star-Shaped Polydimethylsiloxanes

**Scheme 4:**
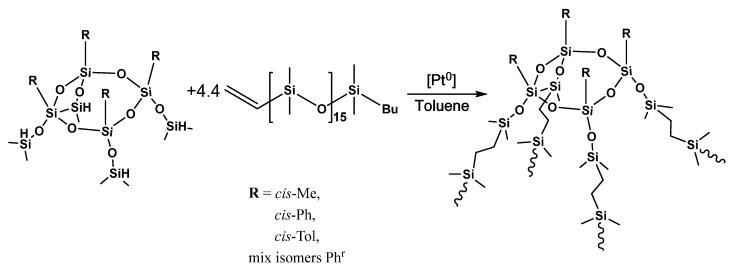
The scheme of **SSP** synthesis on the basis of tetra[organo(dimethylsiloxy)]cyclotetrasiloxane.

For **SSP** synthesis on the basis of various tetracyclic cores, the reaction of hydrosilylation was carried out in the presence of Karsted’s catalyst in toluene (Scheme 4).

The course of the reaction was monitored by ^1^H NMR based on the disappearance of SiH signals in the initial organocyclosiloxane. Four **SSPs** with identical quantity and length of arms but with different cores were synthesized as a result. Molecular-mass characteristics of the polymers obtained are presented in Table 1. All polymers have narrow molecular-mass distribution.

### 3.4. Thermal Properties

The synthesized **SSP** and the initial arm (**PDMS-15**) were studied by TGA and DSC methods.

**Figure 4:**
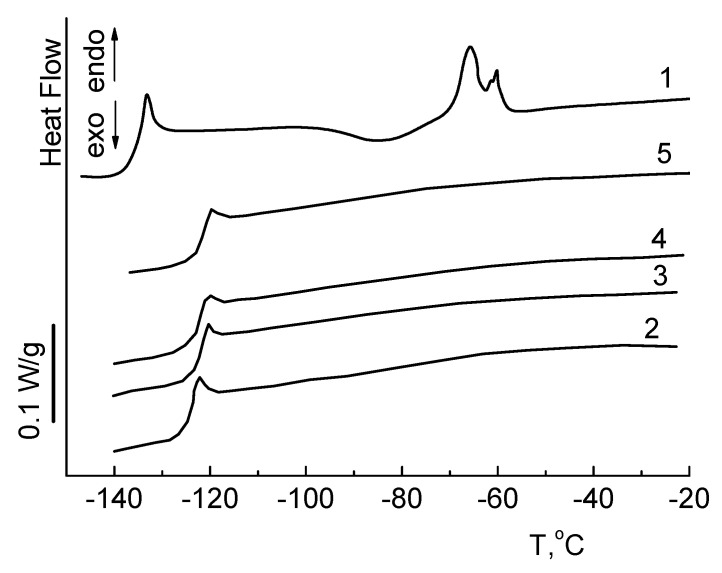
DSC curves for **PDMS-15** (1) **and Me_4_-15** (2), **Tol_4_-15** (3), **Ph_4_-15** (4), **Ph^r^_4_-15** (5) star-shaped polymers at 10 °C/min heating rate.

In Figure 4, DSC curves for **Ph^r^_4_-15, Me_4_-15, Ph_4_-15 и**, and **Tol_4_-15** polymers differing in organic substituent at Si atom and stereoregularity of the cyclic core, (**Ph^r^_4_-15** and **Ph_4_-15** polymers) and initial **PDMS-15** are presented. According to DSC data obtained, full suppression of PDMS-arm crystallization process is observed in all **SSPs**. Similarly, crystallization of side chains was not observed for SSPs with 21 PDMS units per arm [44]. Thus, introduction of a cyclic fragment as the branching core suppresses the ability of polymeric chains of the target products to crystallize.

Glass-transition temperatures for all **SSPs** are close and are within −124 to −122 °C that is characteristic of classical linear PDMS [51].

**Figure 5:**
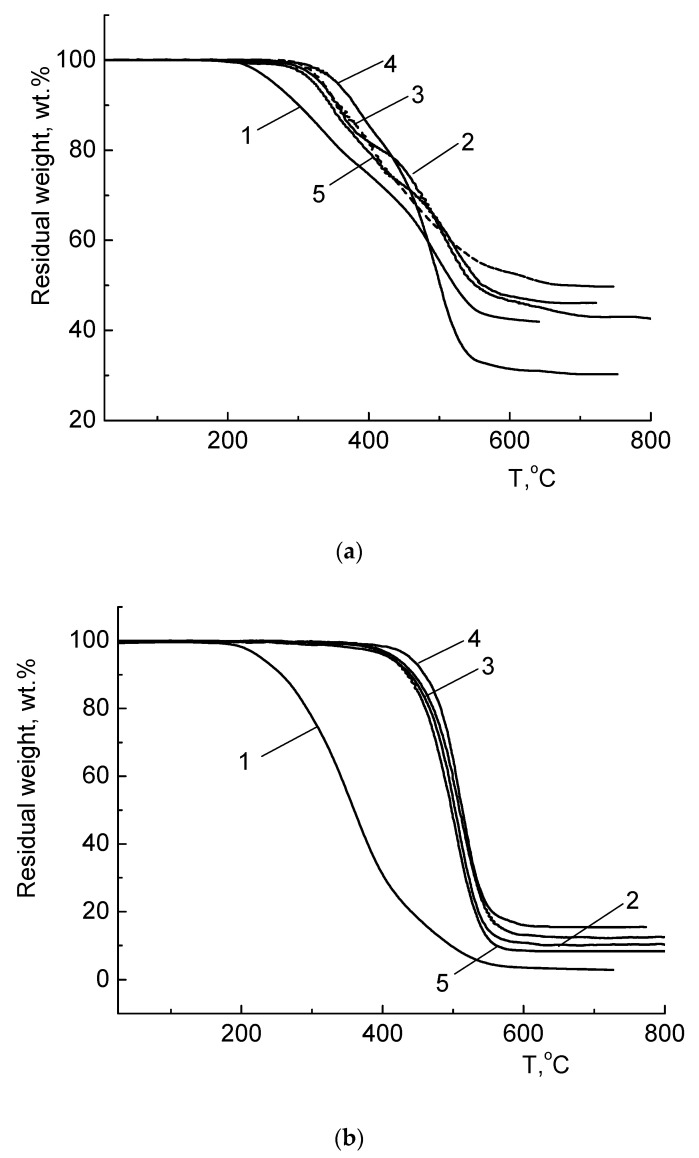
TGA curves for **PDMS-15** (**1**) and **Ph_4_-15** (**2**), **Ph^r^_4_-15** (**3**), **Tol_4_-15** (**4**), **Me_4_-15** (**5**) star-shaped polymers both in air (**a**) and in argon (**b**) at 10 °C/min heating rate.

TGA data obtained for **Ph^r^_4_-15**, **Me_4_-15**, **Ph_4_-15**, and **Tol_4_-15** polymers and **PDMS-15** arm are presented in Table 2 and in Figure 5. They show **SSP** improved thermal and thermo-oxidative stability compared with their linear arm. The destruction onset temperatures in argon and in air are within temperature limits typical for linear PDMS of similar molecular weight [51].

### 3.5. Rheological Properties

#### In Solution

Intrinsic viscosity [η] depends on solvent quality, i.e., on its thermodynamic affinity to polymer. The macromolecular coil in various solvents swells differently. The “better” is the solvent the bigger is its size that, in turn, results in bigger hydrodynamic resistance to flow and [η] increase. Heptane is a “good” solvent for PDMS that is confirmed by value χ = 0.409 defined in [52]. In [53], the constants of Mark–Kuhn–Houwink equation for linear PDMS in heptane at 25 °C [η] = 1.207 × 10^−4^ M^0.741^ were found.

**Figure 6:**
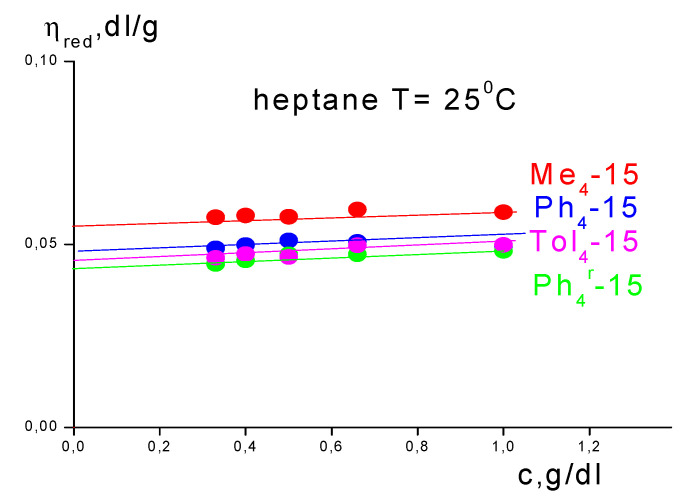
Concentration dependences of reduced viscosity of dilute solutions of star-shaped **Ph^r^_4_-15**, **Me_4_-15**, **Ph^r^_4_-15** and **Tol_4_-15** in heptane.

The measured values of intrinsic viscosity for **SSP** and calculated values of intrinsic viscosity of their linear analogs are presented in Table 3 and Figure 6.

In Figure 6, concentration dependencies of reduced viscosity of diluted solutions in heptane are presented. The values of intrinsic viscosity [η] defined at C → 0 are given in Table 3.

As presented data show (Table 3 and the Figure 6), all **SSPs** have small intrinsic viscosity values irrespective of macromolecule branching-out center structure that might evidence rather dense **SSP** macromolecule packing in solution.

The results of **SSP** rheological study in bulk are presented in Table 3. Low viscosity values in both solution and block are characteristic of practically all studied samples, and still viscous flow activation energy values “feel” the cyclic branching-out center. For all studied samples, viscous flow has high power consumption.

**Figure 7:**
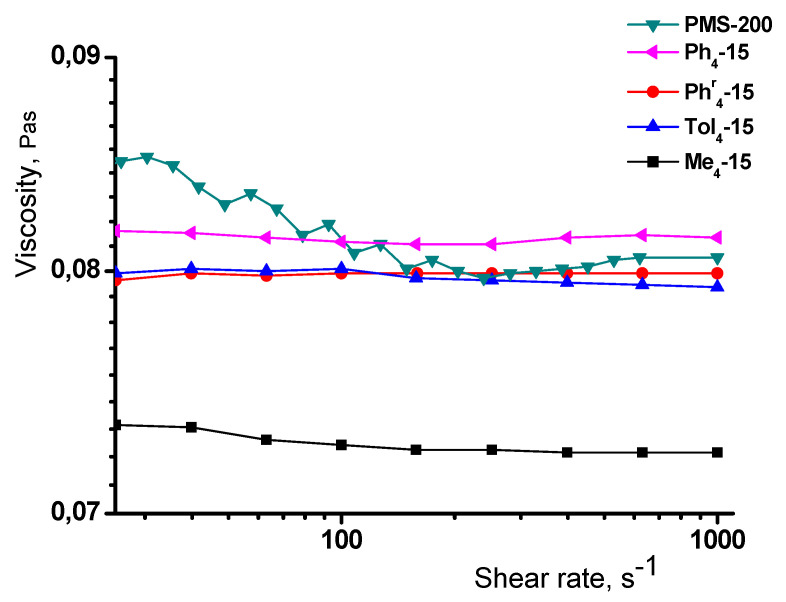
**Ph^r^_4_-15, Me_4_-15, Tol_4_-15, Ph_4_-15**, and **PMS-200** flow curves at 20 °C.

Flow curves of **SSP** and of their linear analog **PMS-200** are presented in Figure 7. As Figure 7 shows, **SSP** viscosity practically does not depend on shift rate that indicates the Newtonian character of the flow.

For linear polymer, the Newtonian flow begins at shear rates over 100 s^−1^. At smaller shift rates, viscosity decreases as the shift rate grows that is related to gradual orientation of polymer macromolecules in the shear field. **SSP** viscosity in the whole interval of shear rates is less than for linear polymer, and **Me_4_-15** has the least viscosity among **SSPs**.

**Figure 8:**
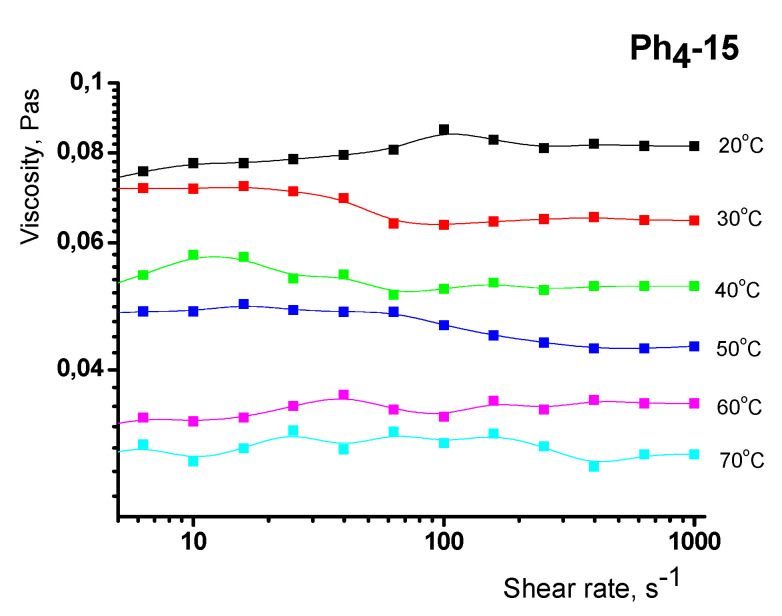
Flow curves of star-shaped **Ph_4_-15** polymer at 20, 30, 40, 50, 60, and 70 °C.

Flow curves of **Ph_4_-15** at various temperatures are presented in Figure 8. Similar dependencies were also obtained for other **SSPs**.

**Figure 9:**
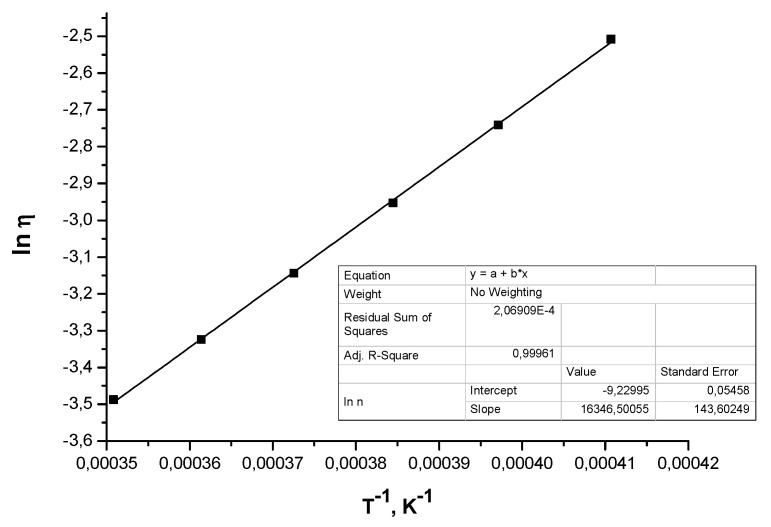
Defining E_a_ for **Ph_4_-15** polymer.

The viscosity temperature dependencies in Arrhenius equation coordinates (Figure 9) were plotted. 

They have linear shape that allows to calculate viscous flow activation energy (E_a_) for all **SSPs**.

Based upon the data obtained, it can be assumed that the macromolecule coil size in bulk is smaller than for the polydimethylsiloxane analog. On the other hand, as PDMS-arms have much smaller length, they orient quicker in the shift field that is confirmed by the Newtonian character of their flow at all shear rates.

### 3.6. Langmuir Layers

Amphiphilic siloxane **SSPs** form Langmuir monolayers at the air–water interface after spreading of the solutions and evaporation of the solvent. The macromolecules contain identical hydrophilic fragments and differ by hydrophobic substituents. Hydrophilic covalent-ionic Si-O bonds are directed into water subphase and form hydrogen bonds with water molecules. Hydrophobic butyl-, methyl-, phenyl-, or tolyl- groups are directed into air phase. **SSPs** studied in this paper differ from their analogs with bigger number of arms [44]. They have strictly four arms of 15 Si-O units instead of 21. Their cores contain both similar [44] phenyl-groups, and methyl- or tolyl- groups. Such SSP selection allows the discovery of the following: (1) whether SSP behavior in Langmuir monolayers is influenced by the type of hydrophobic groups in the core (among **Me_4_-15**, **Ph_4_-15**, and **Tol_4_-15**); (2) how regular cis-SSP features manifest themselves in comparison with their irregular analog, while all their other molecular parameters are identical (**Ph^r^_4_-15**, and **Ph_4_-15**).

**Figure 10:**
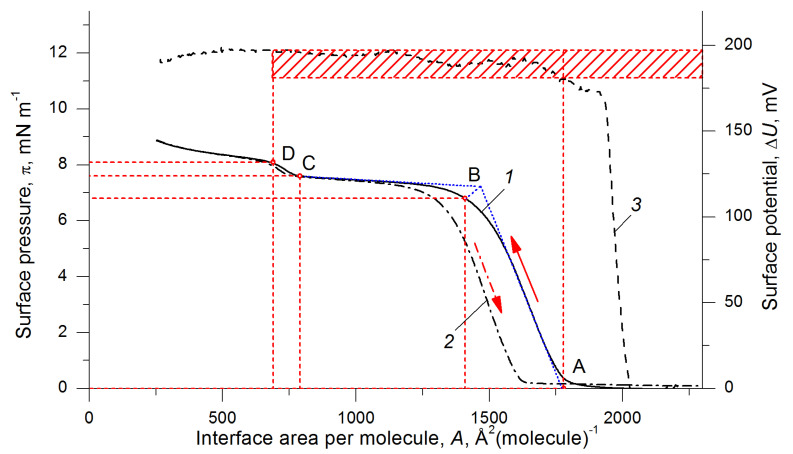
π-A (**1**,**2**) and ΔU-A (**3**) compression (**1**,**3**) and expansion (**2**) isotherms of **Me_4_-15** Langmuir layer. T = 20 °C. Letters A–D denote characteristic points on the surface pressure isotherm.

**Figure 11:**
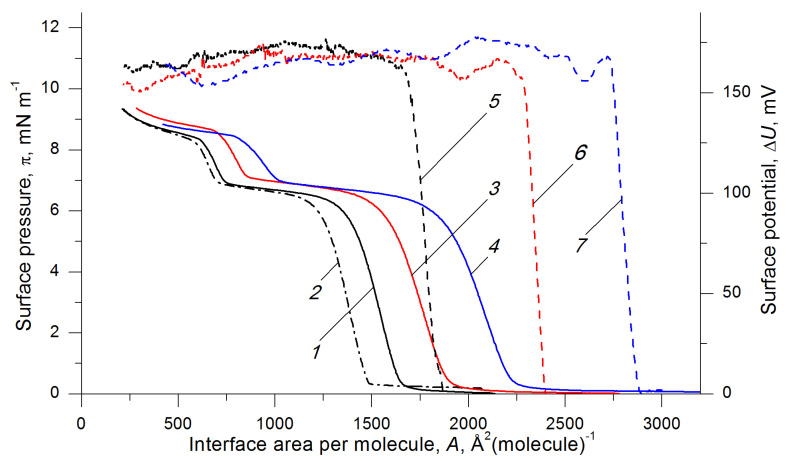
π-A (**1**–**4**) and ΔU-A (**5**–**7**) compression (**1**,**3**–**7**) and expansion isotherms (**2**) of **Ph^r^_4_-15** (**1**,**2**,**5**), **Ph_4_-15** (**3**,**6**), and **Tol_4_-15** (**4**,**7**) Langmuir layers. T = 20 °C.

**Figure 12:**
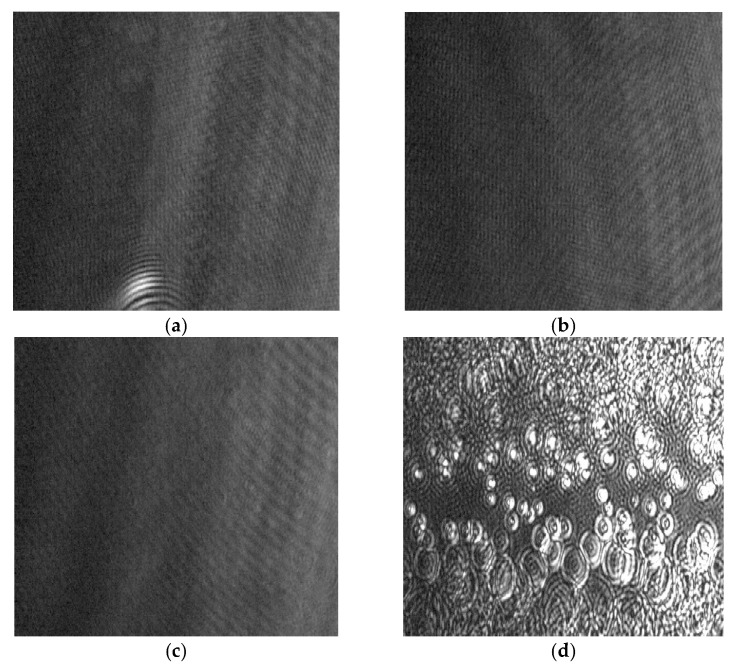
Brewster angle microscopy images of **Me_4_-15** Langmuir layer surface obtained at compression up to the following values of surface pressure and area per a molecule: 0 mN m^−1^ and 1890 Å^2^ (**a**), 3.8 mN m^−1^ and 1610 Å^2^ (**b**), 7.5 mN m^−1^ and 1030 Å^2^ (**c**), 8.4 mN m^−1^ and 490 Å^2^ (**d**).

Dependencies of surface pressure (π) and surface potential (ΔU) on the area of interphase surface per molecule (A) for **Me_4_-15** are given in Figure 10, for **Ph^r^_4_-15**, **Ph_4_-15,** and **Tol_4_-15** in Figure 11. A–D points on curve 1 in Figure 10 correspond to traditional [54,55,56] designation of π-A isotherm characteristic points of PDMS. Values of surface pressure, surface areas both per molecule and per repeating dimethylsiloxane unit in points A–D, and surface potential ranges (defined similarly as shown for curve 1 in Figure 10) are summarized in Table 4. Calculation of changes in surface areas per repeating dimethylsiloxane unit allows **SSPs** to be compared with each other, with PDMS, and with **SSPs** [44]. Brewster angle microscopy images for **SSP** Langmuir layers are shown in Figure 12.

**Me_4_-15** contains butyl groups at the ends of arms as well as methyl groups as substituents at Si atoms similarly to PDMS. The shape of π-A isotherm and the range of surface pressure change between the points C and D (0.5 mN m^−1^) for **Me_4_-15** are similar to the ones observed for linear PDMS. In Brewster angle microscopy images in the area of zero surface pressure (Figure 12a), the border of darker water surface, and lighter **Me_4_-15** monolayer, is visible. At the same time, prior to surface pressure rise, the surface potential isotherm exhibits a jump from zero to positive ΔU values in a narrow interval of surface area change (curve 3 in Figure 10). On compression of a monolayer in the A–B region of π-*A* isotherm (curve 1 in Figure 10), the water surface is completely covered with a monolayer (Figure 12b); the conformational transition in siloxane arms in the B–C region of the *π-A* isotherm does not change Langmuir monolayer surface morphology (Figure 12c). Two hypotheses [57] of conformational transformations of siloxane chain in the B–C region of π-A isotherm of PDMS are known: the formation of horizontal folds from odd quantity of chain segments, or a helix with six dimethylsiloxane units per one turn (analog of Damaschun helicoid at crystallization). However, the surface pressure values in **Me_4_-15** collapse point are lower than those typical for PDMS [57,58]. It can result from both **Me_4_-15** rather low molecular mass as well as formation of less stable layer due to formation of helices consisting of only two coil turns assumed by each **SSP** arm, or steric difficulties caused by attachment of one of PDMS-arms end to the central cycle.

After the final collapse of Langmuir layer in point D (Figure 12d), **Me_4_-15** excess accumulates into lenses, similar to [44]. Brewster angle microscopy image shows bright domains on a dark surface. In the range A–D, surface potential fluctuates irrespective of conformational transformations of the siloxane chain and Langmuir film collapse (curve 3 Figure 10).

An increase in size of organic substituents attached to the core, in **Ph_4_-15**, **Ph^r^_4_-15**, and **Tol_4_-15** results in surface pressure decrease in points B and C of π-A isotherm together with its increase in point D in comparison with **Me_4_-15** (Figure 10 and Figure 11). At the same time, the surface-pressure jump between points C and D increases to 1.4 mN m^−1^ for **Ph_4_-15**, **Ph^r^_4_-15,** and **Tol_4_-15**. It exceeds the range of 0.9 mN m^−1^, which was typical for **Ph_4_-15** analog with arm length of 21 dimethylsiloxane units [44]. Morphological changes in Langmuir monolayers of **Ph_4_-15, Ph^r^_4_-15,** and **Tol_4_-15** polymers according to Brewster angle microscopy are similar to presented for **Me_4_-15** in Figure 12. The surface potential obtained on compression of **Ph_4_-15**, **Ph^r^_4_-15**, and **Tol_4_-15** in the A–D region fluctuates in the range of 150–180 mV, which is reduced in comparison to 180–200 mV typical for linear PDMS and **Me_4_-15** polymer.

The shape of surface pressure isotherms obtained on expansion of **Me_4_-15** and **Ph^r^_4_-15** Langmuir layers (curves 2 in Figure 10 and Figure 11) are similar to those obtained under compression. The hysteresis in compression–expansion cycle is observed in the whole range of surface areas. After Langmuir layers expansion, the excess of polymers collapsed in lenses spreads in a monolayer with conformational transformations of helix or folded chains into straightened ones. The π-A and ΔU-A isotherm shapes, as well as the change in Langmuir layers surface morphology in compression–expansion cycle, indicate their liquid aggregate state.

## 4. Conclusions

The isomerization of *cis*-tetra[(phenyl)(dimethylsiloxane)]cyclotetrasiloxane was carried out for the first time and the mix of all four isomers in equal quantities was obtained with 90% yield. Four new narrow-dispersity non-crystallizable star-shaped polydimethylsiloxanes were synthesized. Their molecules contain identical number of arms of identical length, but have differences in the branching-out center. *Cis*-tetratolyl-, *cis*-tetraphenyl-, and *cis*-tetramethylsilsesquioxane cycles serve as the branching-out center in three **SSPs**, respectively. One **SSP** has the mix of four stereoisomers as a core. **SSP** viscosimetric research showed that their macromolecules are small-size dense coil in both solution and in bulk.

**SSP** study in Langmuir layers at the air–water interface showed that the increase in the size of organic substituents in cyclic core is a major factor for increased stability of Langmuir layer before a collapse. Thus, **Me_4_-15** polymer forms less stable Langmuir layers by 1 mN m^−1^ than PDMS due to macromolecule structure change from linear to star-shaped and smaller molecular weight. Replacement of only four methyl groups in a **SSP** cyclic core to phenyl or tolyl without considerable change in molecular-mass characteristics, strikingly changes the form of the surface pressure isotherm and increases the stability of the layer by 0.5 mN m^−1^.

At the same time, as the general tendency in behavior of studied **SSP,** we note the determinative influence of PDMS-arms in comparison with the features of the branching-out center structure. It seems advisable to continue the assessment of influence of various elements of the **SSP** structure with an even bigger reduction in arms length that would allow the influence of branching-out center features on **SSP** properties to be more accurately revealed.

## Figures and Tables

**Table 1:** Molecular-mass characteristics of a linear arm and star-shaped polymers.

Sample	M_n_^NMR^, kDa	M_n_^GPC^, kDa	M_w_^GPC^, kDa	PDI	Output, %
**PDMS-15**	1.3	2.4	2.7	1.13	95
**Ph_4_-15**	5.9	6.7	7.6	1.14	96
**Ph^r^_4_-15**	5.9	6	6.7	1.11	91
**Tol_4_-15**	5.9	7.9	8.9	1.13	98
**Me_4_-15**	5.5	6.6	7.1	1.08	91

**Table 2:** Thermal characteristics of SSP obtained.

Sample	T_g_, C	T_cc_, C	T_m_, C	T_d_^5%^, C		M, Mas. %	
				Air	Argon	Air	Argon
**PDMS-15**	−133	−83	−60	234	281	43	4
**Ph_4_-15**	−123	-	-	331	410	43	11
**Ph^r^_4_-15**	−122	-	-	337	420	50	13
**Tol_4_-15**	−124	-	-	357	439	20	16
**Me_4_-15**	−123	-	-	322	413	46	9

**Table 3:** Rheological properties of **SSP** in solution and in bulk.

Sample	Star/ Linear Polymer	Pa*s Star	E_a_, kJ/mol
**Ph_4_-15**	0.049/0.091	0.081	16.3
**Ph^r^_4_-15**	0.044/0.083	0.080	16.6
**Tol_4_-15**	0.047/0.100	0.080	16.3
**Me_4_-15**	0.054/0.086	0.073	15.8

**Table 4:** Characteristic points A–D of π-A and ΔU-A isotherms for **Me_4_-15**, **Ph_4_-15**, **Ph^r^_4_-15**, **Tol_4_-15**.

		A	B	C	D
**PDMS** [57,58]	Area per (OSi(CH_3_)_2_) unit, Å^2^	19.0	15.0	8.0	7.0
	Surface pressure, mN m^−1^	0	7.0	8.5	9.0
	Surface potential range, mV	190–200			
**Me_4_-15**	Area per a molecule, Å^2^	1780	1410	790	690
	Area per (OSi(CH_3_)_2_) unit, Å^2^	18.5	14.6	8.2	7.2
	Surface pressure, mNm^−1^	0	6.8	7.6	8.1
	Surface potential range, mV	180–195			
**Ph_4_-15**	Area per a molecule, Å^2^	1900	1530	850	700
	Area per (OSi(CH_3_)_2_) unit, Å^2^	18.5	14.9	8.3	6.8
	Surface pressure, mN m^−1^	0	6.2	7.2	8.6
	Surface potential range, mV	150–175			
**Ph^r^_4_-15**	Area per a molecule, Å^2^	1680	1340	750	610
	Area per (OSi(CH_3_)_2_) unit, Å^2^	18.6	14.8	8.3	6.7
	Surface pressure, mN m^−1^	0	6.1	7.0	8.4
	Surface potential interval, mV	160–175			
**Tol_4_-15**	Area per a molecule, Å^2^	2230	1800	1010	800
	Area per (OSi(CH_3_)_2_) unit, Å^2^	18.6	14.9	8.4	6.6
	Surface pressure, mN m^−1^	0	6	7.0	8.4
	Surface potential interval, mV	155–180			

## Data Availability

Not applicable.
